# Pharmacokinetics and Enterohepatic Circulation of 2-(Quinoline-8-carboxamido)benzoic Acid (2-QBA) in Mice

**DOI:** 10.3390/pharmaceutics16070934

**Published:** 2024-07-12

**Authors:** Ji-Hyeon Jeon, So-Yeon Jeon, Yeon-Ju Baek, Chan-E Park, Min-Koo Choi, Young Taek Han, Im-Sook Song

**Affiliations:** 1 BK21 FOUR Community-Based Intelligent Novel Drug Discovery Education Unit, Vessel-Organ Interaction Research Center (VOICE), College of Pharmacy and Research Institute of Pharmaceutical Sciences, Kyungpook National University, Daegu 41566, Republic of Korea; yoummseggh@knu.ac.kr; 2 College of Pharmacy, Dankook University, Cheon-an 31116, Republic of Korea; jeonsy@dankook.ac.kr (S.-Y.J.); yeonju4227@dankook.ac.kr (Y.-J.B.); 72230411dku@dankook.ac.kr (C.-E.P.); minkoochoi@dankook.ac.kr (M.-K.C.)

**Keywords:** 2-(quinoline-8-carboxamido)benzoic acid (2-QBA), liquid chromatography–tandem mass spectrometry, pharmacokinetics, enterohepatic circulation

## Abstract

The quinoline alkaloid 2-(quinoline-8-carboxamido)benzoic acid (2-QBA), which is isolated from *Aspergillus* sp. SCSIO06786, a deep sea-derived fungus, has been suggested as a therapeutic candidate for the treatment of Parkinson’s disease. We developed an analytical method for 2-QBA using a liquid chromatography–tandem mass spectrometry (LC-MS/MS) in mouse plasma, in which a protein precipitation method for the sample preparation of 2-QBA in mouse plasma was used due to its simplicity and good extraction recovery rates (80.49–97.56%). The linearity of the calibration standard sample, inter- and intraday precision and accuracy, and stability of three quality control samples were suitable based on the assessment criteria and the lower limit of quantification (LLOQ) of the 2-QBA was 1 ng/mL. A pharmacokinetic study of 2-QBA was performed in mice divided into oral (2.0, 5.0, and 15 mg/kg) and intravenous (0.5 and 1.0 mg/kg) administration groups. The absolute oral bioavailability (BA) range of 2-QBA was calculated as 68.3–83.7%. Secondary peaks were observed at approximately 4–8 h after the oral administration of 2-QBA at all doses. The elimination half-life of the orally administered 2-QBA was significantly longer than that of the intravenous 2-QBA. In addition, glucuronide metabolites of 2-QBA were identified. They were transformed into 2-QBA using the β-glucuronidase treatment. Furthermore, the 2-QBA was readily absorbed from the jejunum to lower ileum. Taken together, the secondary peaks could be explained by the enterohepatic circulation of 2-QBA. In conclusion, the reabsorption of orally administered 2-QBA could contribute to the high oral BA of 2-QBA and could be beneficial for the efficacy of 2-QBA. Moreover, the simple and validated analytical method for 2-QBA using LC-MS/MS was applied to the pharmacokinetic study and BA assessments of 2-QBA in mice and would be helpful for subsequent pharmacokinetic studies, as well as for evaluations of the toxicokinetics and pharmacokinetic–pharmacodynamic correlation of 2-QBA to assess its potential as a drug.

## 1. Introduction

Parkinson’s disease (PD) is a progressive neurodegenerative disorder characterized by the loss of dopaminergic (DA) neurons in the substantia nigra of the midbrain, resulting in motor symptoms, such as rest tremors, rigidity, bradykinesia, and a stooped posture [1]. Drugs such as levodopa, selegiline, and amantadine have been used to treat patients with PD; however, they relieve the symptoms rather than directly targeting the causative protein [2,3,4]. The limitations of the current PD therapies highlight the need for developing newer and more effective drugs that interfere with the progression of neurodegenerative processes [1].

Serotonin (5-hydroxytryptamine; 5-HT) plays an important role in physiological, cognitive, and emotional processes. Impairment of the serotonergic system is associated with psychiatric disorders, such as anxiety and depression, and neurological disorders, such as PD, Alzheimer’s disease, and epilepsy [5]. Based on studies reporting that 5-HT6 receptor antagonists can improve cognitive and memory functions, intepiridine, also known as 3-(phenylsulfonyl)-8-(1-piperazinyl)-quinoline, has been studied as a therapeutic drug targeting schizophrenia or Alzheimer’s disease [6]. Intepiridine monotherapy did not lead to significant improvement of mild-to-moderate Alzheimer’s disease; however, as an adjunct to donepezil, intepiridine was associated with sustained improvements in cognition and Alzheimer’s disease symptoms [6,7,8]. A structural analogue, GSK215083 ([N-methyl]3-[(3-fluorophenyl)sulfonyl]-8-(4-methyl-1-piperazinyl) quinoline), is also a quinoline-based compound with a high affinity for the 5-HT6 receptor and has been developed as a positron emission tomography (PET) radioligand for the in vivo measurement of 5-HT6 receptor availability to monitor the acetylcholinergic and dopaminergic neurotransmission and cognitive disorders [5,9,10]. The intravenous injection of [C^11^]GSK215083 showed a substantial distribution in the brain with the rank order of striatum > cortex > cerebellum and an elimination half-life range of 60–90 min. The [C^11^]GSK215083 in the plasma was rapidly eliminated with an elimination half-life range of 10–20 min but accumulated in the lungs, liver, and stomach, which could be vulnerable to radiation exposure [11,12].

**Figure 1:**
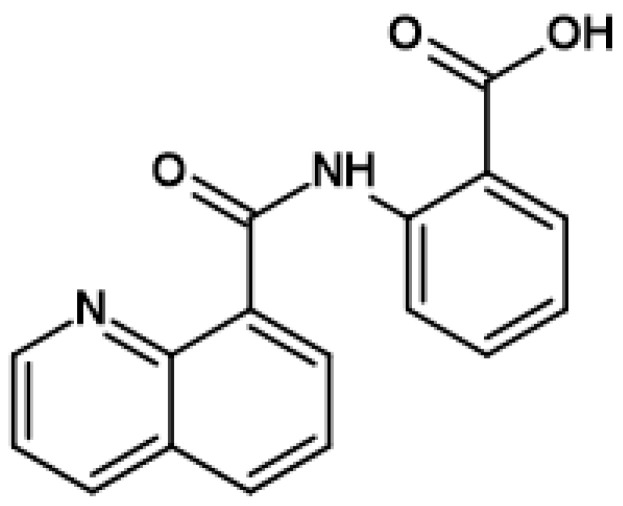
Chemical structure of 2-(quinoline-8-carboxamido)benzoic acid (2-QBA).

Other natural quinoline derivatives have recently drawn researchers’ attention for being scaffolds in medicinal chemistry and advantageous over other molecular scaffolds because of their simple and eco-friendly synthesis methods [13]. For instance, 8-hydroxyquinoline is used as a versatile chelating agent and pesticide precursor [14]. The drugs that have an 8-substituted quinoline moiety such as chloroquine, oxyqunoline, ciprofloxacin, nedocromil, and phenylquiniline-8-carboxamide exert antibacterial, antiviral, anticonvulsant, antispasmodic, and anticancer effects [11,15,16]. Compounds of 8-substituted quinolines have also antioxidant and antipsychotic effects [13]. For example, 2-(quinoline-8-carboxamido)benzoic acid (2-QBA) (Figure 1) is a new quinoline alkaloid isolated from *Aspergillus* sp. SCSIO06786, a deep sea-derived fungus. It is effective for its antioxidant and neuroprotective activity [17]. It also contains an N-acyl anthranilate structure, which is a putative neuroprotective moiety [1]. 2-QBA significantly ameliorates 1-methyla-4-phenylpyridinium ion (MPP+)-induced DA neuronal degeneration in a dose-dependent manner and functionally restored posterior deirids in the range of 2.5–10 μM [1]. It also caused enhanced proteasome activity and accelerated degradation of abnormal α-synuclein protein and had protective potential against α-synuclein neurotoxicity [1,17].

Although the cause of PD is unknown, the loss of DA neurons in the basal ganglia is considered one of the primary causes. Degenerative DA neurons are associated with the abnormal expression of α-synuclein [18,19]. α-Synuclein is a presynaptic neuronal protein that is linked genetically and neuropathologically to PD [20]. Therefore, α-synuclein represents a valid therapeutic target in PD and drug candidates targeting α-synuclein, such as anle138b (3-(1,3-benzodioxol-5-yl)-5-(3-bromophenyl)-1H-pyrazole), ENT-01 (a synthetic squalamine salt), glycerol phenylbutyrate, hypoestoxide, and posiphen in clinical trials [21]. Anle138b and ENT-01 showed promising achievements such as favorable safety and pharmacokinetics profiles and the improvement of cognition and psychosis [22,23,24]. Moreover, the percentage of clinical trials targeting α-synuclein increased from 6.8% during the period of 2020–2022 to 10% in 2023 [21,25]. In this sense, 2-QBA improves the neuronal morphology in MPP+-induced DA neuronal degeneration and also suppresses α-synuclein expression. Therefore, the potential of 2-QBA as a therapeutic candidate for the treatment of PD has been evaluated [1,19].

Despite the expected treatment effects of 2-QBA, bioanalyses using liquid chromatography–tandem mass spectrometry (LC–MS/MS) and pharmacokinetic studies of 2-QBA have rarely been conducted. Considering the physicochemical properties of 2-QBA (i.e., the ClogP, pKa, and water solubility values are 3.57, 3.38 (predicted using ChemDraw Pro 17; CambridgeSoft Corporation, Cambridge, MA, USA), and 35.60 ± 2.71 μg/mL (*n* = 4), respectively), 2-QBA is a lipophilic compound with low aqueous solubility; therefore, it may not have favorable pharmacokinetic properties [26,27]. Thus, herein, we optimized and developed the LC–MS/MS conditions for quantifying 2-QBA in a biological matrix and applied them to investigate the pharmacokinetic properties of 2-QBA in mice.

## 2. Materials and Methods

### 2.1. Chemicals and Reagents

First, 2-QBA (Figure 1) with a purity rate > 99.0% was synthesized according to Lee et al.’s method [1]. The berberine chloride, an internal standard (IS), activated charcoal, β-glucuronidase from Helix pomatia (containing 300 KU β-Glucuronidase and 10 KU sulfatase/g), and sodium acetate were purchased from Sigma-Aldrich (St. Louis, MO, USA). The acetonitrile and water were purchased from Tedia (Fairfield, CT, USA). All solvents and chemicals were of high-performance liquid chromatography (HPLC) and reagent grade.

### 2.2. Development of 2-QBA Analysis Using LC-MS/MS

#### 2.2.1. Instrument Conditions

The Agilent Infinity 1260 Infinite II HPLC system (Agilent Technologies, Santa Clara, CA, USA) was used as the LC system and Synergi Polar RP (150 × 2.0 mm, 4 μm; Phenomenex, Torrance, CA, USA) was used for chromatographic separation. A mixture of 70% acetonitrile containing 0.1% formic acid and 30% water containing 0.1% formic acid (pH 2.7) was used as the mobile phase with isocratic elution. The flow rate of the mobile phase was set to 0.3 mL/min and the column temperature was maintained at 30 °C.

Agilent 6470 triple-quadrupole MS with an electrospray ionization source (Agilent Technologies, Santa Clara, CA, USA) was used for the mass spectrometric detection and quantitative analysis. The mass spectrometer was operated in the positive ion mode, with multiple reaction monitoring (MRM) transitions at *m*/*z* 293.1→155.9 for 2-QBA and at *m*/*z* 336.1→320.0 for berberine (IS), with optimized fragmentor rates of 100 eV and 30 eV, respectively.

#### 2.2.2. Preparation of Stock and Working Solutions of 2-QBA

For preparation, the 2-QBA was dissolved in acetonitrile at a concentration of 1 mg/mL as the stock solution. The stock solution was diluted to prepare the working solution for calibration standards and quality control (QC) with acetonitrile. The concentrations were 1, 2, 5, 20, 50, 250, and 500 ng/mL for the calibration standards and 3, 100, and 400 ng/mL for the QC samples. terberine was dissolved in acetonitrile at a concentration of 1 ng/mL and used as the IS.

Aliquots of calibration standards and QC samples (30 μL) were evaporated with nitrogen gas and the residue was reconstituted with 30 μL of blank mouse plasma. The final concentrations were 1, 2, 5, 20, 50, 250, and 500 ng/mL for the calibration standards and 3 (low QC), 100 (middle QC), and 400 (high QC) ng/mL for the QC samples.

#### 2.2.3. Sample Preparation

One hundred and twenty microliters of IS solution (berberine 1 ng/mL in acetonitrile) was added to 30 μL aliquots of the calibration standards, QC samples, and plasma samples of 2-QBA. The mixture was vigorously vortexed for 5 min and centrifuged at 13,000× *g* for 5 min. The supernatant was transferred to an autosampler vial and a 3 μL aliquot was injected into the LC-MS/MS system.

### 2.3. Validation of Analytical Method Validation

The analytical method was validated in terms of its selectivity, linearity, inter- and intraday accuracy and precision, stability in three environments, matrix effects, and recovery according to the US Food and Drug Administration (FDA) Guideline for Bioanalytical Methods (accessed on 14 June 2023; https://www.fda.gov/regulatory-information/search-fda-guidance-documents/bioanalytical-method-validation-guidance-industry).

#### 2.3.1. Selectivity and Linearity

To estimate the selectivity, a chromatogram of blank plasma from mice was compared with that of each blank plasma sample from mice containing lower limit of quantification (LLOQ) samples (1 ng/mL of 2-QBA) and the IS.

The calibration standard curve for 2-QBA (1–500 ng/mL) was plotted to the concentrations of 2-QBA versus the ratio of the peak areas of 2-QBA and the IS. The linearity of the standard curve was assessed using a least-squares linear regression analysis.

#### 2.3.2. Intra- and Interday Precision and Accuracy

To determine the interday precision and accuracy, two sets of QC samples (3, 100, and 400 ng/mL) were tested for five independent days. In the case of the intraday precision and accuracy estimations, six sets of QC samples (3, 100, and 400 ng/mL) were analyzed on the same days. The coefficient of variance (CV, %) was calculated to determine the precision and the accuracy of the analysis was evaluated based on the percentage of the measured QC concentration to the nominal QC concentration.

#### 2.3.3. Matrix Effect and Recovery

The matrix effect is expressed as the percentage of the peak area from the post-extraction method, which was extracted first before adding two QC samples (concentrations of 3 and 400 ng/mL), in regard to the peak area from the equivalent concentration of the neat solution. The matrix effect of the IS was tested using the same methods as the procedure for 2-QBA, using a 1 ng/mL concentration of the IS.

The extraction recovery was estimated by comparing the peak area from the pre-extraction samples, which were extracted after adding two QC samples (concentrations of 3 and 400 ng/mL), to the post-extraction samples, with corresponding concentrations as described above. The extraction recovery of the IS was tested using the same methods as the procedure for 2-QBA, using a concentration of 1 ng/mL of IS.

#### 2.3.4. Stability

The stability of 2-QBA in mouse plasma was tested in terms of its benchtop stability, freeze–thaw stability, and autosampler stability. In all stability tests, three sets of QC samples (3, 100, and 400 ng/mL) were prepared.

The benchtop stability was tested by incubating the samples at 25 °C for 6 h. For the freeze–thaw stability, the samples were placed at −80 °C for over 12 h for freezing and thawed at 25 °C for 6 h as one cycle; the same protocol was repeated for three cycles. Finally, the autosampler stability was tested by placing the prepared QC samples in the autosampler at 6 °C for 24 h. After analyzing all of the stability test samples, the concentrations were calculated using freshly prepared calibration standards.

#### 2.3.5. Dilution Integrity

Aliquots (30 μL) of 2-QBA working solution (4 μg/mL and 2 μg/mL in acetonitrile) were evaporated under nitrogen gas flow and the residues were reconstituted with 30 μL of blank mouse plasma, followed by 10- and 5-fold dilutions with blank mouse plasma to yield a final concentration of 400 ng/mL 2-QBA. Three sets of diluted 2-QBA samples (10- and 5-fold) were analyzed using freshly prepared calibration standards and the precision and accuracy of the diluted samples were computed, as described for the intra- and interday precision and accuracy.

### 2.4. Dose-Dependent Pharmacokinetics of 2-QBA

Male ICR mice (7 weeks old, 35–30 g; Samtako, Osan, Korea) were acclimated to the animal facility at Kyungpook National University for one week with free access to animal chow and water. In the per oral administration (PO) groups, the mice were fasted for 14–16 h prior to the administration of 2-QBA.

For intravenous (IV) administration, 2-QBA (0.5 mg/kg in 3 mL of saline containing 10% DMSO) was injected via the tail vein for eight mice that were randomly divided into two groups for sparse sampling. Blood samples were collected via the jugular vein at 0.083, 0.5, 2, and 8 h from 4 mice and at 0.25, 1, 4, and 24 h from another 4 mice. Eight mice were administered 1 mg/kg of 2-QBA using the same protocol described above.

After the PO administration of 2-QBA (2 mg/kg in 5 mL of saline containing 10% DMSO) using an oral gavage for 10 mice, blood samples were collected at 0.25, 0.75, 2, and 8 h from 5 mice and at 0.5, 1, 4, and 24 h from another 5 mice. Ten mice in each group were administered 5 mg/kg or 15 mg/kg of 2-QBA using an oral gavage and blood was drawn using the same protocol described above. All blood samples were centrifuged at 13,000× *g* for 1 min and the supernatant plasma (30 μL) was stored at −80 °C until the analysis of the 2-QBA. For the analysis of 2-QBA in the plasma samples, 120 μL of IS solution (berberine 1 ng/mL in acetonitrile) was added to plasma samples (30 μL). The mixture was vigorously vortexed for 5 min and then centrifuged at 13,000× *g* for 5 min. The supernatant was transferred to autosampler vials. Subsequently, a 3 μL aliquot was injected into the LC-MS/MS system.

We also investigated the excretion profile of 2-QBA after IV and PO administration at doses of 1 mg/kg for IV and 2 mg/kg for PO. After IV or PO administration of 2-QBA, the mice were returned to a metabolic cage to collect the urine and feces samples. The urine and feces samples were collected during the periods of 0–24 h, 24–48 h, 48–72 h, and 72–96 h. The urine ball was washed using equal volumes of methanol and added to the urine samples. The fecal samples were homogenized with 9 volumes of water to yield 10% fecal homogenates. The aliquots (30 μL) of urine and 10% fecal homogenate samples were stored at −80 °C until the analysis of the 2-QBA. For the analysis of the 2-QBA, 120 μL of IS solution was added to the 30 μL aliquot of urine and 10% fecal homogenate samples. The mixture was vigorously vortexed for 5 min and then centrifuged at 13,000× *g* for 5 min. The supernatant was transferred to autosampler vials. Subsequently, a 3 μL aliquot was injected into the LC-MS/MS system.

### 2.5. Effect of Activated Charcoal on the Pharmacokinetics of 2-QBA

To elucidate the reabsorption mechanism of 2-QBA, a pharmacokinetic study of 2-QBA was conducted with activated charcoal (AC) administered PO. Male ICR mice (7 weeks old, 25–30 g) were fasted for 14–16 h prior to the administration of 2-QBA.

In total, 28 mice were administered 2-QBA (1 mg/kg/3 mL) via the tail vein. Two hours after the 2-QBA dosing, AC (0.8 g/kg/5 mL in distilled water) or a vehicle (5 mL/kg distilled water) was administrated via oral gavage. Each group included ten mice who were randomly divided into two groups for sparse sampling. Blood collection was performed at 0.083, 0.5, 2, and 8 h from 5 mice and at 0.25, 1, 4, and 24 h from another 5 mice, as described previously. Four mice treated with either AC (0.8 g/kg) or the vehicle 2 h after the 2-QBA IV dosing (1 mg/kg) were returned to a metabolic cage to collect the urine and feces samples. The urine and feces samples were collected for 72 h.

Next, 2-QBA was administered PO (2 mg/kg/5 mL) with or without AC (0.8 g/kg in 3 mL of DW), which was administered via oral gavage 2 h after the 2-QBA dosing. Each group included eight mice who were randomly divided into two groups for sparse sampling. Blood collection was performed at 0.25, 0.75, 2, and 8 h from 4 mice and at 0.5, 1, 4, and 24 h from another 4 mice, as described previously. Four mice treated with either AC (0.8 g/kg) or vehicle 2 h after the 2-QBA PO dosing (2 mg/kg) were returned to a metabolic cage to collect the urine and feces samples. The urine and feces samples were collected for 72 h.

Aliquots of plasma samples (30 μL) were added to 120 μL of IS solution. The mixture was vigorously vortexed for 5 min and then centrifuged at 13,000× *g* for 5 min. The supernatant was transferred to autosampler vials. Subsequently, a 3 μL aliquot was injected into the LC-MS/MS system. Aliquots (30 μL) of urine and 10% feces homogenate samples were added to 60 μL of 150 mM sodium acetate buffer (pH 5.0) in the presence or absence of β-glucuronidase (900 unit) at 37 °C for 2 h. The reaction was quenched by adding four volumes of ice-cold IS solution. The mixture was vigorously vortexed for 5 min, followed by centrifugation at 13,000× *g* for 5 min. The supernatant was transferred to autosampler vials and subsequently a 3 μL aliquot was injected into the LC-MS/MS system [26,28].

### 2.6. Identification of Glucuronide Metabolites of 2-QBA

An Agilent Infinity 1260 Infinite II HPLC system equipped with Agilent 6470 triple-quadrupole MS (Agilent Technologies, Santa Clara, CA, USA) was used to elucidate glucuronide metabolites of 2-QBA. Synergi Polar RP (150 × 2.0 mm, 4 μm; Phenomenex, Torrance, CA, USA) was used for the chromatographic separation. A mixture of 70% acetonitrile containing 0.1% formic acid and 30% water containing 0.1% formic acid was used as the mobile phase with isocratic elution. The flow rate of the mobile phase was set to 0.15 mL/min and the column temperature was maintained at 30 °C.

The mass spectrometer was operated in the positive ion mode, with MRM transitions at *m*/*z* 293.1→156.1 for 2-QBA (optimized fragmentor of 110 V; collision energy of 20 eV), at *m*/*z* 469.1→293.1 for 2-QBA-glucuronide (2-QBA-Glu) (140 V; 15 eV), and at *m*/*z* 336.1→320.0 for berberine (IS) (135 V; 30 eV).

### 2.7. Intestinal Permeability of 2-QBA

The small intestines were isolated from fasted mice under isoflurane anesthesia (1–3% with oxygen gas flow) and dissected into three parts, the jejunum, upper ileum, and lower ileum. All parts of the small intestines were cut vertically, washed twice with Hank’s Balanced Salt Solution (HBSS; pH 7.4), mounted on the tissue holder of a Navicyte Easy Mount Ussing Chamber (Warner Instruments, Holliston, MA, USA), and acclimated in HBSS (pH 7.4) for 15 min with continuous oxygenation (95% O_2_/5% CO_2_). The intestinal permeability of 2-QBA was assessed by adding 0.5 mL of HBSS containing 20 μM of 2-QBA on the donor side and 0.5 mL of fresh HBSS on the receiver side. Then, a 100 μL aliquot was withdrawn from the receiver side every 30 min for 2 h and an equal volume of pre-warmed fresh HBSS was added to replenish the lost volume. To analyze the 2-QBA, 100 μL aliquots were mixed with 4 volumes of IS solution (1 ng/mL berberine in acetonitrile). The mixture was vigorously vortexed for 5 min and then centrifuged at 13,000× *g* for 5 min. The supernatant was transferred to autosampler vials. Subsequently, a 3 μL aliquot was injected into the LC-MS/MS system. To confirm the feasibility of the permeability study in intestinal segments, four permeability marker compounds, namely caffeine, propranolol (for high permeability), ofloxacin (for moderate permeability), and atenolol (for low permeability), were used for a positive control study, as previously described [29].

### 2.8. Data Analysis and Statistics

WinNonlin 5.1 software (Pharsight Co., Mountain View, CA, USA) was used to calculate the pharmacokinetic parameters. AUC_last_ and AUC_inf_ indicate the areas under the plasma concentration curve from 0 h to the last sample and from 0 h to infinity, respectively; these were estimated using the linear trapezoidal method. T_1/2_ is the elimination half-life, which is estimated from the elimination coefficient (k) (i.e., 0.693/k), and MRT is the mean residence time. CL is the total clearance, which is calculated by dividing the dose by AUC_inf_. Vdss is the volume of the distribution at a steady state, calculated from MRTxCL. CL_renal_ and CL_feces_ are calculated by dividing the amount excreted in urine and feces by the AUC_inf_, respectively. CL_metabolism_ is calculated by subtracting CL_renal_ and CL_feces_ from CL. C_max_ and T_max_ are the maximum plasma concentration and time to reach C_max_, respectively. BA is calculated by dividing the dose-normalized AUC_inf_ for PO administration (AUC_inf_/PO dose) by the dose-normalized AUC_inf_ for IV administration (AUC_inf_/IV dose). All data are expressed as the mean ± standard deviation (SD).

## 3. Results

### 3.1. LC-MS/MS Analysis of 2-QBA

#### 3.1.1. Optimization of Analysis

**Figure 2:**
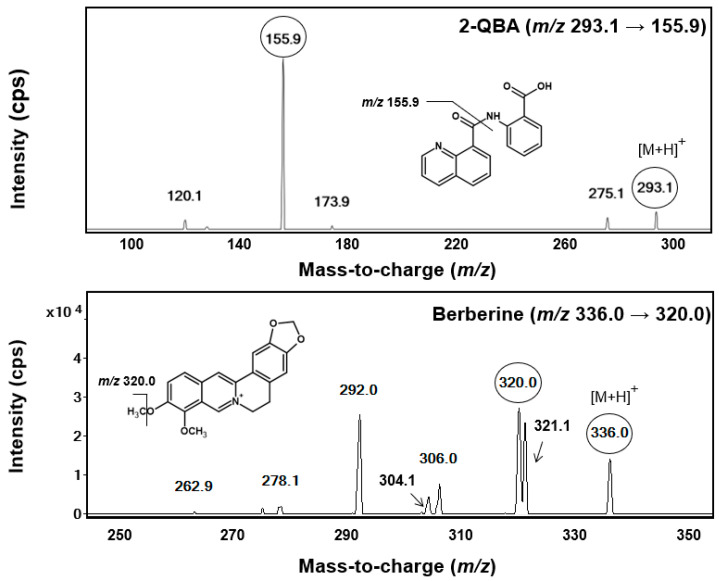
Product ion scan of 2-QBA and berberine (IS).

First, we optimized the electrospray ionization conditions for the 2-QBA and the IS. Both compounds were appropriately ionized in positive mode. The MRM transition of 2-QBA was selected as the precursor ion ([M+H]^+^, *m*/*z* 292.9) and the most abundant product ion (*m*/*z* 156.0) (Figure 2). The MRM transition of berberine (IS) was used as the precursor ion ([M+H]^+^, *m*/*z* 336.0) and the product ion (*m*/*z* 320.0) (Figure 2), which was the same condition used previously [26,30].

**Figure 3:**
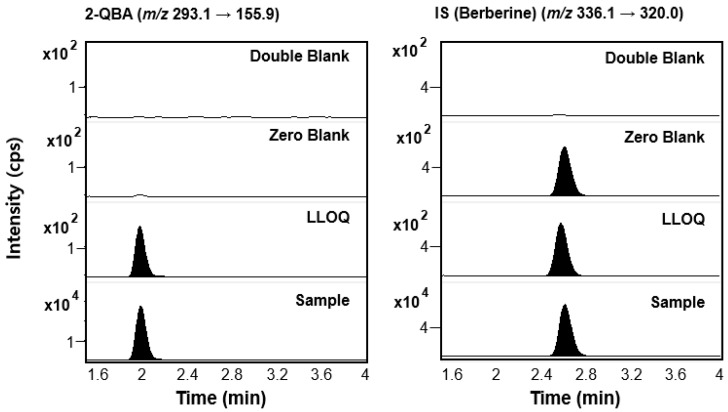
Representative MRM chromatograms of 2-QBA and IS (berberine) in mouse plasma for the double-blank, zero-blank, LLOQ (1 ng/mL), and plasma samples at 0.25 h following PO administration of 2-QBA.

Several quinoline and isoquinoline derivatives were tested as the IS for the quantification of 2-QBA. Among these, berberine was selected as the IS in this study because it shows good and reproducible extraction recovery and matrix effect results and sufficient peak intensity after protein precipitation using acetonitrile. Additionally, the peak of berberine in the chromatogram was completely separated from that of 2-QBA within the short time range of 1.8–2.8 min. As a result, each chromatographic run was completed within 4 min (Figure 3).

The following step involved testing various mobile phases and columns to detect and quantify 2-QBA adequately in the biological samples. The sensitivity and peak shape were better in acetonitrile than in methanol for the analysis of a wide range of 2-QBA; therefore, isocratic elution of 70% acetonitrile was finally selected based on the peak shape and retention time of 2-QBA. In the column study, a Kinetex C18 column (75 × 4.6 mm, 2.6 μm), Hydro-RP (100 × 2.0 mm, 2.5 μm), and Synergi Polar RP (150 × 2.0 mm, 4 μm) were compared. The sensitivity and peak reproducibility were better with the Synergi Polar RP column than with the Kinetex C18 and Hydro-RP column.

The representative MRM chromatograms of 2-QBA and berberine from double-blank, zero-blank, calibration standard (LLOQ 1 ng/mL), and plasma samples at 0.25 h after PO administration are shown in Figure 3. The selectivity was further confirmed in male ICR mouse plasma samples of six different origins, and the results showed no disturbance peaks derived from the mouse blank plasma for the retention times of 2-QBA and the IS under our MS/MS analysis conditions. The retention times for 2-QBA and the IS were observed at 2.0 and 2.6 min, respectively. The signal-to-noise (S/N) ratio was calculated for the validity and suitability of the LLOQ of 2-QBA compared with the blank samples and was appropriate at >10.0 (Figure 3). Finally, the eluted peaks of 2-QBA and berberine showed good selectivity without interference around the retention time.

#### 3.1.2. Linearity

The calibration standard curve of 2-QBA showed linearity in the concentration range of 1–500 ng/mL, with a coefficient of determination (r^2^) for linear regression curves of 0.997 and accuracy and precision rates of less than 15% (Table 1).

#### 3.1.3. Precision and Accuracy

The intra- and interday precision and accuracy of 2-QBA assessed using three levels of QC samples are presented in Table 2. The intra- and interday precision ranged from 1.22 to 6.98% for 2-QBA and the intra- and interday accuracy ranged from 100.38 to 108.86%, which satisfied the acceptance criteria (i.e., 85–115%) according to the US FDA Guideline for Bioanalytical Method Validation.

#### 3.1.4. Matrix Effect and Recovery

The matrix effects and extraction recovery rates of 2-QBA were tested using two levels of QC samples (Table 3). The matrix effects of the sample preparation method for 2-QBA ranged from 56.47 to 69.00%, with CV values of 11.1% for low QC and 8.74% for high QC. These results suggested that the co-eluting the substances showed substantial matrix effects on the ionization of the analytes of 2-QBA. However, the matrix effects were stable in terms of QC for the six samples of each plasma matrix. Therefore, we concluded that our sample preparation process can be used to analyze the concentrations of 2-QBA in mouse plasma samples. The extraction recovery rates were high and reproducible in the range of 80.48–91.71%, with a CV range of 3.42–7.56%, showing the suitability of our sample preparation method. The extraction recovery and matrix effects of the IS were also high and reproducible (Table 3).

#### 3.1.5. Stability

The precision and accuracy rates for 2-QBA for the three different conditions of stability testing were estimated (Table 4). Under three different conditions of stability testing, the accuracy rates ranged from 89.14 to 109.52% and the precision rates ranged from 2.49 to 13.20%, demonstrating that 2-QBA was stable in all conditions tested. In other words, 2-QBA was stable up to 6 h at 25 °C on the benchtop, for 24 h in the autosampler after sample preparation, and during three freeze–thaw cycles.

#### 3.1.6. Dilution Integrity

The dilution integrity of 2-QBA was assessed by calculating the precision and accuracy from three samples at 4000 ng/mL and 2000 ng/mL. The precision and accuracy of the samples diluted by 10- and 5-fold were assessed (Table 5). The precision rates were 4.66% and 1.53%, respectively. The accuracy rates ranged from 94.16 to 96.49%, which also satisfied the acceptance criteria (i.e., 85–115%).

### 3.2. Pharmacokinetics of 2-QBA

**Figure 4:**
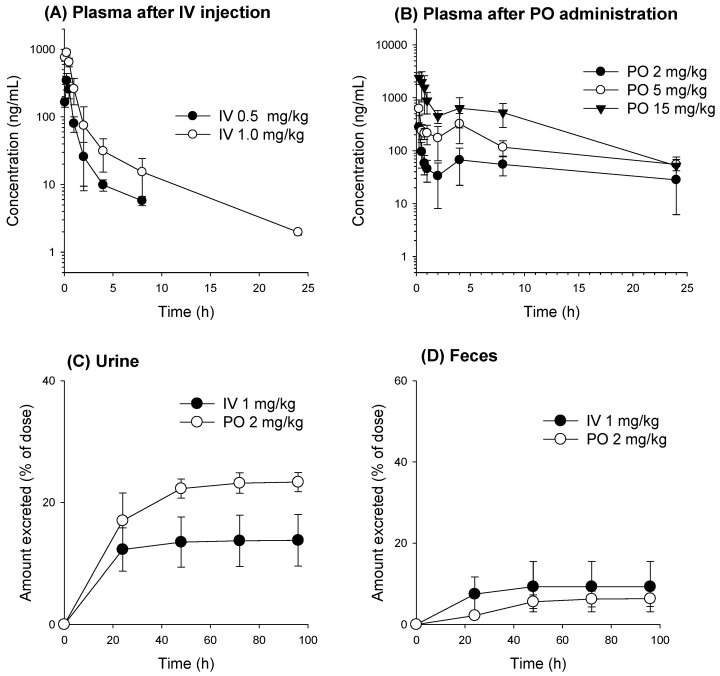
Plasma concentration–time profiles of 2-QBA in mice after (**A**) IV injection of 2-QBA at doses of 0.5 and 1.0 mg/kg (*n* = 4) and (**B**) single PO administration of 2-QBA at doses of 2, 5, and 15 mg/kg (*n* = 5). (**C**) Urinary and (**D**) fecal excretion in mice after IV injection (IV 1 mg/kg, *n* = 4) and PO administration (PO 2 mg/kg, *n* = 3) of 2-QBA, respectively. Data are represented as the means ± SD.

The plasma concentration–time profile of 2-QBA and the respective pharmacokinetic parameters after IV and PO administration are shown in Figure 4 and Table 6.

At first, the 2-QBA was administered intravenously at doses of 0.5 mg/kg and 1.0 mg/kg. The AUC values of the 2-QBA increased proportionally to the doses without significant changes in the elimination half-life (T_1/2_), mean residence time (MRT), clearance (CL), and volume of distribution (Vdss) (*p* > 0.05 in Table 6). In addition, the 2-QBA in the plasma underwent two-phase exponential decay, suggesting a fast distribution phase and moderate elimination phase. Consistent with this finding, the 2-QBA showed a high Vdss. To elucidate the elimination process of 2-QBA in mice, we measured the recovery of 2-QBA from urine and feces samples collected for 96 h using a metabolic cage. The excreted amount of 2-QBA as the parent form into urine and feces is shown in Table 6.

The total recovery rate of 2-QBA over 96 h collected as the parent form was 13.8 ± 4.24% after IV dosing (1.0 mg/kg), and the amount of 2-QBA recovered in the urine was 26.2-fold greater than that recovered from the feces. Therefore, 2-QBA seems to be excreted mainly via the urinary route as the parent form in mice. The calculated renal clearance (CL_renal_), fecal clearance (CL_feces_), and metabolic clearance (CL_metabolism_) rates of 2-QBA were 5.47 ± 2.22, 0.32 ± 0.23, and 10.5 ± 1.31 mL/min/kg, respectively. These results suggest that 2-QBA shows two major elimination pathways; 2-QBA undergoes renal excretion as the parent form and is metabolized prior to being excreted.

When 2-QBA was administered PO at a dose of 2 mg/kg, the plasma profile seemed to undergo the re-absorption process at approximately 5.60 ± 2.19 h according to the basis of the second peak (T_max,2_) after the fast first absorption phase (T_max,1_ at 0.20 ± 0.11 h) (Figure 4B and Table 7). When 2-QBA was given PO at doses of 2, 5, and 15 mg/kg to mice, the dose-normalized AUC values of 2-QBA were not statistically significantly different among the three dose groups. This suggests that the oral AUC increased proportionally to the dose. The oral BA range was calculated as 68.3–83.7%, suggesting favorable intestinal absorption of 2-QBA.

However, the T_1/2_ and MRT values obtained from PO doses from the 2, 5, and 15 mg/kg groups were significantly different, which could be attributed to the significantly shorter T_1/2_ and MRT in mice administered 15 mg/kg than in those administered 2 mg/kg and 5 mg/kg PO. The T_1/2_ and MRT values following PO administration of all three doses were significantly greater than the T_1/2_ values after IV injection (Table 6 and Table 7). In addition, the total amount of 2-QBA excreted via the renal route for 96 h after PO dosing was greater than that after IV dosing. Considering the unabsorbed 2-QBA in the case of PO dosing, the greater urinary excretion of 2-QBA following PO dosing than IV dosing suggests the recycling of 2-QBA from the excreted 2-QBA metabolites. The consistent presence of a second peak and higher T_1/2_ and MRT values for 2-QBA following PO dosing than following IV dosing also suggest the possibility of the enterohepatic circulation of 2-QBA in mice.

### 3.3. Effect of AC on the Pharmacokinetics of 2-QBA

**Figure 5:**
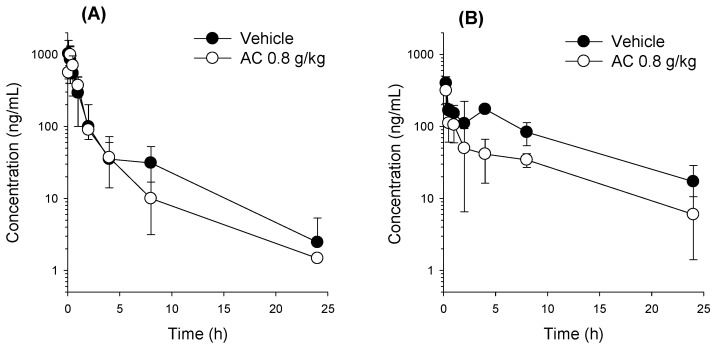
Plasma concentration–time profiles of 2-QBA in mice treated with or without AC after (**A**) IV dosing of 2-QBA (1.0 mg/kg) and (**B**) PO dosing (2 mg/kg). Data are represented as the means ± SD (*n* = 5 for IV, *n* = 4 for PO).

To investigate whether the reabsorption of 2-QBA could occur, we used an AC treatment 2 h after the administration of 2-QBA. AC has been used to prevent the intestinal reabsorption of drugs [31,32]. The plasma concentration–time profiles and pharmacokinetic parameters after the IV and PO administration of 2-QBA with or without AC post-treatment are shown in Figure 5 and Table 8, respectively.

The AC treatment tended to decrease the plasma concentration of 2-QBA after 4 h but did not alter the pharmacokinetics parameters of 2-QBA when administered intravenously (Figure 5A and Table 8). However, we should note that the second peak was observed (vehicle group) after IV administration, differing from Figure 4A.

The AC treatment reduced the plasma concentrations of the PO-administered 2-QBA, and the second peak observed at approximately 4–5 h disappeared (Figure 5B). As a result, the AC treatment significantly decreased the AUC_24h_ and AUC_24h_ values of 2-QBA without significant alterations to the C_max_, T_max_, T_1/2_, and MRT. The urinary and fecal excretion of the 2-QBA administered intravenously did not differ based on whether AC was administered or not. However, the fecal excretion of PO-administered 2-QBA was significantly increased in the AC treatment group compared with the control group (Table 8). The results suggest that the reabsorption of 2-QBA may be disturbed by the presence of AC, and the amount of unabsorbed 2-QBA was consistently increased in the AC treatment group.

### 3.4. Identification of Glucuronide Metabolites of 2-QBA

**Figure 6:**
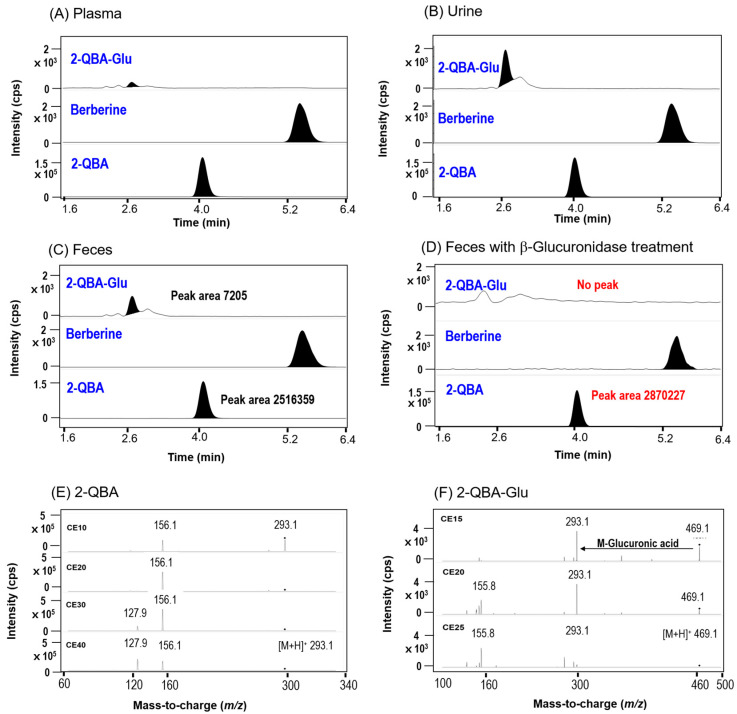
MRM chromatograms of 2-QBA-Glu, berberine (IS), and 2-QBA in (**A**) plasma, (**B**) urine, and (**C**) fecal homogenate samples following the PO administration of 2-QBA. (**D**) Fecal homogenate samples were incubated with β-glucuronidase at 37 °C for 1 h. Product ion scan of (**E**) 2-QBA and (**F**) 2-QBA-Glu identified from fecal homogenate samples with increasing CE values.

To elucidate the reabsorption mechanism of 2-QBA, we identified the glucuronide metabolites of 2-QBA from plasma, urine, and fecal samples after PO administration (Figure 6A–C). We found that 2-QBA-glucuronide (2-QBA-Glu) was present in the plasma, urine, and fecal samples. A product ion scan of 2-QBA with increasing collision energy values for the peaks at 4.1 min elucidated from fecal samples showed product ions at *m*/*z* 293.1 ([M+H]^+^ ion of 2-QBA), *m*/*z* 156.1 (loss of aminobenzoic acid from 2-QBA), and *m*/*z* 127.9 (Figure 6E), which were identical to the product ions from the authentic standard of 2-QBA (Figure 2). A product ion scan of the peak at 2.9 min showed [M+H]^+^ ions at *m*/*z* 469.1, which is 176 amu more than the [M+H]^+^ ion of 2-QBA (*m*/*z* 293.1), and *m*/*z* 155.8 (loss of aminobenzoic acid from 2-QBA), indicating that the peak at 2.7 min might be 2-QBA-Glu (Figure 6F). Moreover, the amount of 2-QBA was increased and the peak of 2-QBA-Glu almost disappeared after the β-glucuronidase treatment at 37 °C for 2 h (Figure 6C,D), suggesting that incubation with β-glucuronidase degraded the 2-QBA-Glu and transformed it back into 2-QBA, which could be reabsorbed into the systemic circulation.

**Figure 7:**
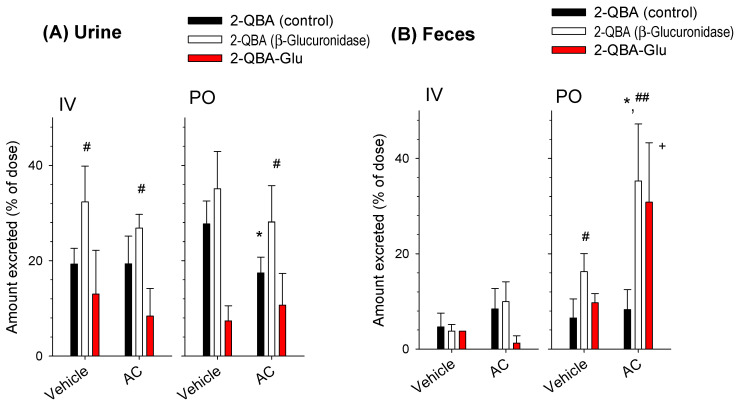
(**A**) Urinary and (**B**) fecal excretion of 2-QBA (■, □) and 2-QBA-Glu (red bar) after IV injection (1 mg/kg) and PO administration (2 mg/kg) of 2-QBA to mice with vehicle or AC (0.8 g/kg) treatment. Data are represented as the mean ± SD (*n* = 4); * *p* < 0.05, statistical significance of 2-QBA between vehicle and AC treatment; # *p* < 0.05, ## *p* < 0.01, statistical significance of 2-QBA between control and β-glucuronidase treatment; + *p* < 0.05, statistical significance of 2-QBA-Glu between control and β-glucuronidase treatment.

Then, we quantified the 2-QBA and 2-QBA-Glu from the urine and fecal samples obtained from the mice after IV or PO administration of 2-QBA. In addition, the amount of 2-QBA-Glu was calculated by subtracting the control 2-QBA from the total 2-QBA with β-glucuronidase treatment. Considerable amounts of 2-QBA and 2-QBA-Glu were excreted into the urine after the IV injection of 2-QBA but the excreted 2-QBA and 2-QBA-Glu were not altered by the AC treatment (Figure 7A). Moreover, the 2-QBA excreted into the feces after the IV injection of 2-QBA was also not affected by the AC treatment (Figure 7B). These results suggest that the 2-QBA and 2-QBA-Glu were mainly excreted into the urine after IV injection; therefore, they were not subjected to the reabsorption process.

The urinary excretion of 2-QBA but not 2-QBA-Glu after the PO administration of 2-QBA was significantly reduced by the AC treatment (Figure 7A); this was attributed to the decreased plasma concentration of 2-QBA in the AC treatment group (Figure 5). Compared with the case of urinary excretion, the fecal excretion of 2-QBA was not affected by the AC treatment but the amount of 2-QBA-Glu was significantly increased in the AC group compared with the vehicle group (Figure 7B). The results suggest that the AC treatment inhibited the transformation of 2-QBA from 2-QBA-Glu and consequently inhibited the reabsorption of 2-QBA from the intestines.

### 3.5. Absorptive Permeability of 2-QBA in Intestinal Segments

**Figure 8:**
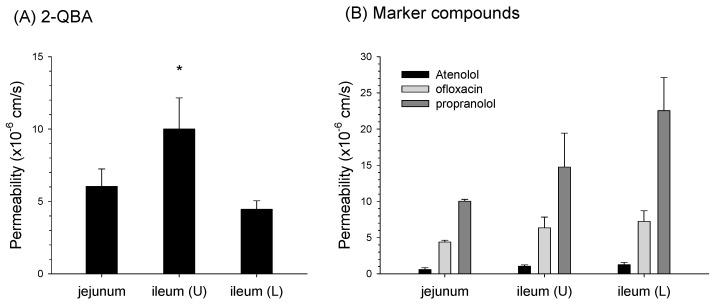
Absorptive permeability of (**A**) 2-QBA and (**B**) marker compounds in intestinal segments. Ileum (U): upper part of the ileum; Ileum (L): lower part of the ileum. Data are represented as the means ± SD (*n* = 4 for 2-QBA and *n* = 3 for marker compounds). * *p* < 0.05, statistically significant from the Kruskal-Wallis test and subsequent post hoc analysis.

The absorptive permeability of 2-QBA was the highest in the upper part of the ileum (ileum (U)) and was lower in the jejunum and lower part of the ileum (Figure 8A). The permeability of propranolol, a high permeability marker, was 10.0–22.5 × 10^−6^ cm/s, while that of ofloxacin, a moderate permeability marker, was 4.38–7.23 × 10^−6^ cm/s and that of atenolol, a low permeability marker, was 0.60–1.25 × 10^−6^ cm/s (Figure 8B). Compared with the marker compounds, the absorptive permeability of 2-QBA in the upper ileum was similar to that of ofloxacin, while the rates in the jejunum and lower ileum were similar to that of propranolol, making the absorptive permeability of 2-QBA moderate to high. These results suggest that 2-QBA can be continuously absorbed with moderate to high permeability and that 2-QBA transformed from 2-QBA-Glu can also be reabsorbed in the lower part of the intestines.

## 4. Discussion

With increasing achievements being made in the field of PD research, clinical research statistics from the past three years have been reported. Among 147 clinical trials during the period of 2020–2022, the use of novel candidates, repurposed candidates, and reformulations accounted for 42.2%, 34.0%, and 19.7% of the studies, respectively. However, only 25% of phase 3 clinical trials were studied using novel drug candidates and the other 75% were studies of repurposed candidates and reformulations [21]. The results suggest that novel drug candidates for PD therapeutics are being studied in the early drug development stage or clinical phase.

In this study, 2-QBA was synthesized using two scaffolds of quinoline and N-acyl anthranilate, which have been characterized for their neuroprotective and antipsychotic activity [1]. As the first step to investigate the pharmacokinetic and pharmacodynamic characterization and correlation of 2-QBA, we developed bioanalytical methods for 2-QBA using LC-MS/MS. The developed analytical methods involved the protein precipitation method using acetonitrile and had a total run time of 4 min. The S/N ratio of the LLOQ (1 ng/mL) was calculated as 388; therefore, the detection limit can be lowered if necessary. A bioanalytical method for 2-QBA was developed and fully validated in terms of its linearity, selectivity, accuracy, precision, matrix effect, extraction recovery, stability, and dilution integrity.

This method was applied to the pharmacokinetic investigation of 2-QBA following its IV and PO administration. When administered intravenously, the 2-QBA was mainly eliminated via urinary excretion and metabolism. Considering the glomerular filtration range of a mouse (6–10 mL/min/kg) [33,34,35] and plasma protein binding rate of 2-QBA (87.2 ± 4.16%, *n* = 6; measured in this study), the CL_renal_ of 2-QBA (5.47 ± 2.22 mL/min/kg) demonstrated that it was mainly excreted via glomerular filtration. The plasma profile of 2-QBA administered PO showed multiple peak phenomena, with T_max,1_ at approximately 0.20–0.30 h and T_max,2_ at approximately 3.50–5.60 h. The mean absorption time was 8.02 h, which was calculated from the difference between MRT_PO_ and MRT_IV_ (i.e., MRT_PO_–MRT_IV_) [29]. The absorptive permeability of 2-QBA (i.e., 4.46–10.0 × 10^−6^ cm/s) was estimated to be moderate to high in the jejunum and upper and lower parts of the ileum when compared with the marker compounds. These results collectively suggest that 2-QBA could be readily and continuously absorbed from the jejunum to the lower part of the ileum and consequently enabled the longer absorption time and significantly longer T_1/2_ than for 2-QBA administered intravenously [36,37]. To understand the multiple peak phenomena of 2-QBA when administered PO, we treated mice with high-dose AC after the appearance of the first peak to interrupt the enterohepatic circulation of 2-QBA because AC has the ability to trap chemicals with its highly adsorbent pores and disrupt the intestinal absorption [27]. We treated the mice with AC 2 h after 2-QBA administration according to previously published methods [32,38] and compared the pharmacokinetics of 2-QBA in the AC treatment group with the vehicle (without AC) group. The AC treatment removed the second peak phenomenon and reduced the plasma exposure of the orally administered 2-QBA (Figure 5). The AC treatment also increased the amount of 2-QBA-Glu by 3-fold in the feces following the oral administration of 2-QBA compared with the vehicle group (Figure 7). These results may be attributed to the interruption of the de-glucuronidation of 2-QBA-Glu in the gut and consequently to the inhibition of the 2-QBA’s reabsorption from the lower part of the intestines caused by the adsorption of 2-QBA and 2-QBA-Glu with AC. Moreover, the decreased plasma exposure of 2-QBA in the presence of AC reduces the oral BA of 2-QBA, which in turn reduces the urinary excretion rate of 2-QBA from 27.7% to 17.5% of the administered PO dose (Figure 4 and Figure 7).

In addition to the reabsorption of 2-QBA, administration-route-dependent glucuronidation metabolism and its excretion could be the distinctive features of 2-QBA (Figure 7); that is, the intravenously administered 2-QBA was mainly excreted into urine as its parent form and glucuronide metabolites. Since the fecal recovery rate of 2-QBA and 2-QBA-Glu was less than 5%, the reabsorption and enterohepatic circulation of 2-QBA following an IV injection of 2-QBA was rarely observed. However, the PO-administered 2-QBA was mainly excreted into urine as its parent form but a substantial amount of 2-QBA-Glu was recovered from the feces. The large amount of 2-QBA-Glu could be reabsorbed and introduced into the systemic circulation through the de-glucuronidation of 2-QBA-Glu in the intestine and the permeation as its parent form in the lower ileum. This enterohepatic circulation of 2-QBA can increase the amount of plasma 2-QBA and the excretion of 2-QBA via the renal route, which could contribute to the higher urinary excretion rates of orally administered 2-QBA than for IV doses of 2-QBA (Figure 4C and Figure 7A). This is evidenced by the results showing that the blockade of the de-glucuronidation of 2-QBA-Glu and reabsorption process by the AC treatment reduced the urinary excretion of the parent 2-QBA and produced comparable rates of urinary excretion of 2-QBA to the IV dose (Figure 7A).

A similar phenomenon was also reported for genistein and resveratrol. The urinary recovery rates of genistein following IV and PO administration were 10.6~11.6% and 19.9~23.3% of the dose [39,40]. Glucuronide metabolites of genistein (genistein-Glu) were excreted into both the bile and urine but the urinary excretion of genistein-Glu was 2-fold greater than its biliary excretion. Moreover, multiple peaks appeared within 6 h of the PO administration of genistein at doses of 6.25~50 mg/kg but did not obviously appear after the IV injection of genistein (12.5 mg/kg) [40]. The enterohepatic circulation of genistein through the biliary excretion of genistein-Glu, enzymatic hydrolysis of genistein-Glu to genistein, and intestinal uptake has been proposed to account for the high BA of genistein in rats [41,42]. Resveratrol also showed route-dependent phase II gut metabolism and enterohepatic circulation. As results, it showed a significantly longer T_1/2_ after PO administration than that after IV injection [36]. The resveratrol underwent significant phase II metabolism; therefore, about 25% of the resveratrol glucuronide and sulfate conjugates were excreted into the bile and subjected to enzymatic hydrolysis and reabsorption in the intestine [36,43]. The estimated oral BA rate of resveratrol without the enterohepatic circulation of resveratrol in rats was reduced by about 44.7% compared with the oral BA with the enterohepatic circulation of resveratrol, which supported the important effect of enterohepatic circulation on the oral BA and pharmacokinetic features of resveratrol [36]. In addition, the enterohepatic circulation process for etodolac, ergot alkaloids, genistein, and mycophenolic acid significantly increased the intestinal absorption or prolonged the T_1/2_ following the administration of these drugs either orally or intravenously [42,44,45,46]. However, we should note that the impacts of enterohepatic circulation on the pharmacokinetics and oral BA of drugs or various chemicals may differ depending on the contributions of the hepatic metabolism, renal excretion, and enteric reabsorption processes of these compounds to the overall pharmacokinetic features [37].

## 5. Conclusions

A new and reasonable analytical method for 2-QBA using LC-MS/MS was developed in this study. This method was successfully validated in terms of its precision and accuracy, stability, matrix effect, and extraction recovery ability. It was applied for the pharmacokinetic evaluation and elucidation of the enterohepatic mechanisms of 2-QBA following IV and PO administration. As a result, 2-QBA was found to have linear pharmacokinetic properties in the oral dose range of 2–15 mg/kg. It undergoes enterohepatic circulation after PO administration via the biliary excretion of 2-QBA-Glu, is transformed into 2-QBA in the gut by β-glucuronidase, and is reabsorbed into the systemic circulation based on its moderate to high permeability in the lower ileum. This reabsorption of 2-QBA contributes to its high oral BA rates (68.3–86.6%).

## Figures and Tables

**Table 1:** Calculated concentrations for 2-QBA calibration standards.

Variables	Nominal Concentration (ng/mL)							Slope	r^2^
	1	2	5	20	50	250	500		
Calculated concentration (ng/mL)	1.0	2.0	5.1	19.6	51.8	256	485	0.246	0.997
Accuracy (%)	100.1	100.3	102.8	97.84	103.5	102.3	96.99	-	-
Precision (CV, %)	2.4	4.7	10.2	5.5	3.0	3.7	4.5	14.9	0.09

Data represented as means ± SD (*n* = 5).

**Table 2:** Intra- and interday precision and accuracy of 2-QBA.

	Nominal Concentration (ng/mL)	Measured Concentration (ng/mL)	Precision (%)	Accuracy (%)
Intra-day (*n* = 6)	3	3.11 ± 0.14	4.57	103.56
	100	105.5 ± 6.09	5.77	105.51
	400	401.5 ± 28.0	6.98	100.38
Inter-day (*n* = 5)	3	3.04 ± 0.04	1.22	101.17
	100	108.9 ± 1.91	1.76	108.86
	400	417.0 ± 21.8	5.22	104.24

Data represented as means ± SD.

**Table 3:** Extraction recovery rates and matrix effects for the determination of 2-QBA and IS.

Analyte	QC (ng/mL)	Extraction Recovery (%)	CV (%)	Matrix Effects (%)	CV (%)
2-QBA	3	89.48 ± 6.76	7.56	56.47 ± 6.25	11.1
	400	91.71 ± 3.13	3.42	69.00 ± 6.03	8.74
IS	1	97.46 ± 3.40	3.49	94.64 ± 3.36	3.55

Data represented as means ± SD (*n* = 6).

**Table 4:** Stability of 2-QBA.

Nominal Concentration (ng/mL)	Measured Concentration (ng/mL)	Precision (%)	Accuracy (%)
Bench-top stability (at 25 °C for 6 h)			
3	2.96 ± 0.17	5.61	98.51
100	89.14 ± 8.92	10.0	89.14
400	366.22 ± 13.4	3.66	91.55
Freeze-thaw cycle stability (3 cycles)			
3	3.43 ± 0.26	7.47	114.4
100	104.3 ± 13.8	13.2	104.3
400	425.7 ± 34.3	8.06	106.4
Autosampler stability (at 6 °C for 24 h)			
3	3.29 ± 0.08	2.49	109.5
100	103.1 ± 10.1	9.83	103.1
400	434.7 ± 35.5	8.15	108.7

Data represented as means ± SD (*n* = 3).

**Table 5:** Dilution integrity of 2-QBA.

Nominal Concentration (ng/mL)	Dilution	Measured Concentration (ng/mL)	Accuracy (%)	Precision (%)
4000	10-fold	376.7 ± 17.5	94.16	4.66
2000	5-fold	387.2 ± 4.35	96.49	1.53

Data represented as means ± SD (*n* = 3).

**Table 6:** Pharmacokinetic parameters of 2-QBA after IV administration in mice.

Site	Parameters	Dose		*p* Value
		0.5 mg/kg	1.0 mg/kg	
Plasma	AUC_24h_ (ng·h/mL)	412.3 ± 32.1	996.7 ± 146.6	ND
	AUC_inf_ (ng·h/mL)	417.8 ± 32.3	1044 ± 174.9	ND
	AUC_24h_/dose (ng·h/mL/mg/kg)	824.7 ± 64.1	996.7 ± 146.6	0.083
	AUC_inf_/dose (ng·h/mL/mg/kg)	835.6 ± 64.6	1044 ± 174.9	0.083
	T_1/2_ (h)	4.61 ± 2.19	5.26 ± 1.31	0.773
	MRT (h)	3.88 ± 1.58	3.03 ± 1.11	0.149
	CL (mL/min/kg)	20.03 ± 1.53	16.32 ± 2.81	0.059
	Vdss (L/kg)	4.73 ± 2.15	2.88 ± 0.72	0.153
Excretion	Amount excreted in urine for 96 h (% of dose)	-	13.8 ± 4.24	-
	Amount excreted in feces for 96 h (% of dose)	-	9.30 ± 6.17	-
	CL_renal_ (mL/min/kg)	-	5.47 ± 2.22	-
	CL_feces_ (mL/min/kg)	-	1.59 ± 1.14	-
	CL_metabolism_ (mL/min/kg)	-	9.26 ± 1.43	-

The *p* values were assessed by comparing the two IV dose groups using the Mann–Whitney U-test. ND: not determined. Data represented as means ± SD (*n* = 4).

**Table 7:** Pharmacokinetic parameters of 2-QBA after PO administration in mice.

Parameters	Dose			*p* Value
	2 mg/kg	5 mg/kg	15 mg/kg	
C_max,1_ (ng/mL)	282.6 ± 173.9	752.1 ± 319.3	2457 ± 696.1	ND
T_max,1_ (h)	0.20 ± 0.11	0.25 ± 0.0	0.30 ± 0.11	0.246
C_max,2_ (ng/mL)	141.3 ± 67	194.2 ± 143.6	812.4 ± 221.7	ND
T_max,2_ (h)	5.60 ± 2.19	3.50 ± 1.00	5.60 ± 2.19	0.158
AUC_24h_ (ng·h/mL)	1368 ± 680.5	3514 ± 846.2	10,583 ± 2734	ND
AUC_inf_ (ng·h/mL)	1426 ± 695.9	4369 ± 934.5	11,021 ± 2645	ND
AUC_24h_/dose (ng·h/mL/mg/kg)	684.2 ± 340.3	702.9 ± 169.2	705.5 ± 182.3	0.755
AUC_inf_/dose (ng·h/mL/mg/kg)	712.7 ± 348.0	873.7 ± 186.9	734.7 ± 176.3	0.326
T_1/2_ (h)	7.82 ± 1.76	9.91 ± 1.92	5.58 ± 1.24 *	0.015
MRT (h)	12.0 ± 4.91	13.7 ± 3.14	7.04 ± 0.76 *	0.016
BA (%)	68.3	83.7	70.4	

BA: bioavailability calculated by dividing dose-normalized PO AUC_inf_ and IV AUC_inf_ at a dose of 1 mg/kg; ND: not determined. The *p* values were assessed by comparing three PO dose groups using the Kruskal–Wallis test; * *p* < 0.05, statistically significant from the post hoc analysis. Data are represented as the means ± SD (*n* = 5).

**Table 8:** Pharmacokinetic parameters of 2-QBA with or without AC treatment (0.8 g/kg) after IV and PO administration of 2-QBA in mice.

Administration	Parameters	Treatment		*p* Value
		Control	AC (0.8 g/kg)	
IV (1 mg/kg)	AUC_24h_ (ng·h/mL)	1273.5 ± 360.8	1175.3 ± 264.4	0.637
	AUC_inf_ (ng·h/mL)	1325.8 ± 421.9	1212.5 ± 293.4	0.635
	T_1/2_ (h)	3.88 ± 1.72	3.18 ± 2.52	0.620
	MRT (h)	2.88 ± 1.14	2.48 ± 2.56	0.759
	CL (mL/min/kg)	13.88 ± 5.41	14.50 ± 3.95	0.841
	Vdss (mL/kg)	2.24 ± 0.88	1.89 ± 1.59	0.683
PO (2 mg/kg)	C_max_ (ng/mL)	399.0 ± 90.7	315.2 ± 105.9	0.343
	T_max_ (h)	0.25 ± 0.00	0.25 ± 0.00	1.00
	AUC_24h_ (ng·h/mL)	1932 ± 453.2	649.6 ± 305.4	0.0032
	AUC_inf_ (ng·h/mL)	2114 ± 608.1	835.7 ± 208.9	0.0082
	T_1/2_ (h)	6.05 ± 2.04	5.50 ± 2.24	0.726
	MRT (h)	8.49 ± 3.08	7.00 ± 2.48	0.486
	BA (%)	79.8	34.5	

The *p* values between the control group and AC treatment group were calculated using the Mann–Whitney U-test. Data are represented as the means ± SD (*n* = 5 for IV, *n* = 4 for PO).

## Data Availability

Data are contained within the article.
